# Depression Onset in Long-term Adolescent Cannabinoid Use: A Neurobiological Review

**DOI:** 10.7759/cureus.7759

**Published:** 2020-04-21

**Authors:** Shawn Y Forrester, Nusrat Jahan

**Affiliations:** 1 Psychiatry, California Institute of Behavioral Neurosciences and Psychology, Fairfield, USA; 2 Internal Medicine, California Institute of Behavioral Neurosciences and Psychology, Fairfield, USA

**Keywords:** cannabinoids, depression, adolescence, endocannabinoids

## Abstract

Escalating cannabis use may be linked to decreased motivation and anhedonia, which are symptoms of depression. Adolescent cannabis users with subthreshold depressive symptoms such as reduced motivation may be susceptible to the development of significant anhedonia in addition to impaired emotional development. The main issue regarding depression within the context of cannabis use is whether or not there is a neurobiological basis linking the two variables.

A Medical Subject Headings (MeSH) function PubMed search using the keywords “cannabis, depression, adolescence, endocannabinoid, and temperament” returned 1,109 articles. Data were included from studies that satisfied the following criteria: (i) published within the last 10 years (older studies were included based on relevance), (ii) on adolescent subjects (animal or human), (iii) published in English, (iv) journal articles, systematic reviews, meta-analyses, clinical trials, observational studies (animal or human), (v) on subjects who had unipolar depression with no comorbidities, and (vi) on subjects who used cannabis [with no confounding variables such as the use of ethanol, nicotine, cocaine, lysergic acid diethylamide (LSD), and heroin; and no medical conditions such as comorbid psychosis, mania, or autism].

Depressive symptoms in cannabinoid users were a common co-occurrence partly explained by pleiotropic linkage of genetic locus identified on chromosome 11q23.1-q23.2 and comprises the gene sequence nuclear cell adhesion molecule 1-tetratricopeptide repeat domain 12-ankyrin repeat and kinase domain containing 1-dopamine receptor D2 (NCAM1-TTC12-ANKK1-DRD2). The relationship between the two is not invariant and is influenced by polymorphic DRD, endocannabinoid receptor (CNR), and 5-HT genes. Anhedonia seemed to be the most important symptom. Cannabinoid-induced long-term neuroplastic changes, particularly in the dorsal striatum, is a possible mechanism resulting in anhedonia and long-term effects on motivation.

## Introduction and background

In this era of receding cannabis restrictions, the likelihood of increased access to cannabinoid products of high delta-9-tetrahydrocannabinol (THCΔ9) content creates a potential public health dilemma, especially given the association of cannabinoids with adverse psychosocial outcomes. Human longitudinal studies, while not consistent, have shown a correlation between the early, escalating cannabis use with the onset of depressive symptoms such as anhedonia and decreased social interaction [1-3]. The issue regarding depression within the context of cannabis use is whether or not there is a neurobiological basis linking the two, specifically among adolescents and young adults. Collectively, antecedent observational studies have, at best, suggested a concurrent correlation between the two and a possible predictive relationship. Neurobiological data from animal studies, functional magnetic resonance imaging (fMRI), and genome-wide analysis have also shown a variable correlation between depressive symptoms and cannabinoid exposure in adolescence. This article will review the most recent neurobiological data from animal and genetic studies examining the onset of depression in relation to cannabis use in adolescence.

Method

To facilitate this review, 1,109 articles resulting from a MeSH function PubMed search using the keywords “cannabis, depression, adolescence, endocannabinoid, and temperament” were examined. Articles were included if they satisfied the following criteria: (i) published within the last 10 years (older studies were included based on relevance), (ii) on adolescent subjects (animal or human), (iii) published in English, (iv) journal articles, systematic reviews, meta-analyses, clinical trials, observational studies (animal or human), (v) on subjects who had unipolar depression with no comorbidities, and (vi) on subjects who used cannabis [with no confounding variables such as the use of ethanol, nicotine, cocaine, lysergic acid diethylamide (LSD), and heroin; and no medical conditions such as comorbid psychosis, mania, or autism].

## Review

Adolescent brain development

The adolescent phase of development is characterized by profound neurobiological changes and unprecedented psychosocial stressors. Adolescents transition during this period from the role of dependent minors to the role of adults who are self-directed, independent, problem-solving, and emotionally self-regulated. It is also a time of significant vulnerability to high-risk behaviors, addiction, and the emergence of mental illness. In early adolescence, there is a marked increase in gray matter density attributed to a tremendous rate of synaptogenesis. This especially occurs in the prefrontal cortex (PFC; the area of the brain specializing in planning, decision making, and effortful attention). Animal models demonstrate a subsequent decline in gray matter volume due to gradual synaptic depletion, a process known as “synaptic pruning.” As many as 30,000 synapses per second are lost from both cerebral hemispheres during later adolescent development [4]. This typically occurs in a posterior-to-anterior fashion with the superior temporal cortex being the last to complete this phase of maturation. Subcortical structures such as the hippocampus, amygdala, and the nucleus accumbens complete this pruning process in early adolescence compared to phylogenetically more advanced centers such as the superior temporal cortex and the PFC. In comparison, white matter tracts, as a result of myelin growth and increase in axonal size and caliber, develop simultaneously throughout the cerebrum [5,6]. Like gray matter changes, white matter tracts undergo rapid development in early adolescence, followed by gradual consolidation in later adolescence and into early adulthood. Diffusion tensor imaging suggests that white matter tracts specifically connecting PFCs with basal ganglia structures continue throughout the adolescent period [5,7]. Animal studies using adolescent male rats suggests that the brain, during early adolescence, also undergoes adaptive changes to many neurotransmitter systems. Major changes have been demonstrated specifically in the gamma-aminobutyric acid (GABA), glutamine, and endocannabinoid (eCB) receptor density [8,9].

GABA and glutaminergic development

The secretion of steroid hormones is a hallmark of the onset of puberty, and, hence, the changes in adolescent GABA synaptic function may in part be attributed to the increased levels of steroid hormones. Steroid hormones through direct interaction with GABA receptors influence the numbers of GABA receptors as well as the expression of specific GABA subunits, and this may account for the dramatic changes observed in the function of GABA subtype A (GABA_A_) receptors at the onset of puberty. In animal models, GABA_A _receptor activity changes during adolescence. GABA_A_ typically functions as a depolarizing receptor mediating early postnatal processes such as proliferation and migration of neuronal cells to specialized functional areas and neuronal differentiation to specific circuits [10]. This GABA function remains this way throughout life until the onset of puberty when GABA receptors begin to mediate a hyperpolarizing and inhibitory function [11]. By the time the adolescent becomes an adult, GABA receptors located on interneurons within larger neuronal circuits function predominantly as inhibitors or modulators of output neuronal signals. As with all other neurotransmitter systems, the GABA system undergoes refinement first in phylogenetically primitive areas such as the hippocampus where maturation is achieved before the onset of puberty. However, in higher centers such as the PFC, maturation occurs later. As such, working memory and executive volition, which are strongly associated with GABA activity, reach full maturation in late adolescence at or around the age of 19 years.

In addition to its role in the development of volition and working memory during adolescence, it is suggested that GABA may also play a role in the consolidating of neural circuits and tracts involving conditioned behavioral responses and long-term memory. Looking at the GABA synapses in the cortex, their numbers increase dramatically in terms of total numbers resulting from dendritic growth, arborization, and dendritic-axonal connectivity. The formation of these synapses, like all other synapses in the cortices during adolescence, occurs in the general trend towards excess, followed by synaptic pruning. Interaction between the dopaminergic and the GABA has been shown to be significant. In the PFC, GABA interneurons are strongly innervated by dopaminergic afferents and, hence, GABA synapses are directly modulated by dopamine. GABA modulatory and inhibitory functions are directly influenced by dopamine activity. It is important to highlight that although dopamine receptor subtype 1 (D1) is functional on interneurons independent of the postnatal age, specific stimulation of interneurons via dopamine receptor subtype 2 (D2) receptors is observed only after the 36th postnatal day in rats, indicating that the interaction between these two neurotransmitter systems is altered during adolescence.

Glutaminergic changes

In prepubescent years, GABA receptor-mediated depolarization is associated with enhanced Mg2+ removal from N-methyl D-aspartate (NMDA) receptors [10]. As such, GABA and glutamate activity have significant neuroplastic functions determining long-term potentiation (LTP) or long-term depression (LTD). Both neurotransmitter systems incorporate some levels of postsynaptic glutamate α-amino-3-hydroxy-5-methyl-4-isoxazolepropionic acid (AMPA) receptor subtype 2 (GluR2), an AMPA subtype characterized by divalent ion permeability. These GluR2 subtypes gradually replace non-GluR2 subtypes in addition to more Ca2+ permeable NMDA receptors [12,13]. The later temporal expression of these AMPA subtypes seems to play a role in early oscillatory activity patterns of neuronal circuits and the neuroplastic changes associated with memory retention, neurocognitive permanence, and the general consolidation of neural pathways that characterize this period of development.

Endocannabinoid role in adolescent development

**Figure 1:**
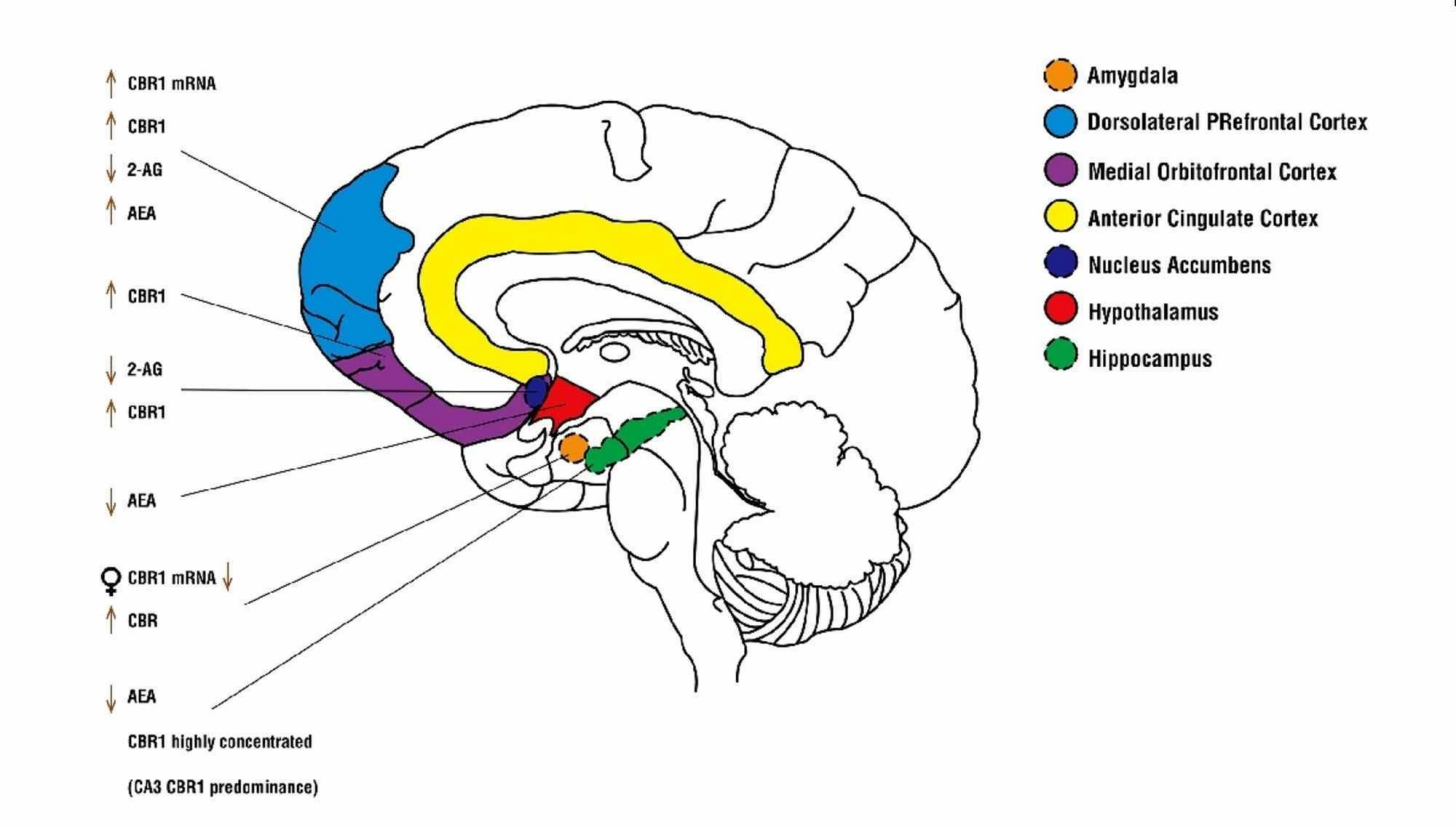
Changes in the endocannabinoid system of the adolescent brain AEA: anandamide; 2-AG: 2-arachidonoylglycerol; CBR1: cannabinoid receptor 1; CA3: dorsal hippocampus; mRNA: messenger ribonucleic acid

The eCB system comprises a very dynamic, poorly understood system of lipid neurotransmitters and receptor types. What is reliably demonstrable are the profound changes occurring during adolescence (Figure 1). Neurotransmitter molecules N-arachidonylethanolamine (AEA) and 2-arachidonoylglycerol (2-AG) regulate synaptic function and neuroplastic changes during adolescence and into adulthood. In regards to the latter process, the eCB plays an integral role in the modulation of GABAergic and glutaminergic neuronal circuits in addition to its regulation of monoamine neural circuit activity. AEA and 2-AG are synthesized from phospholipid precursors and are not constitutionally produced, nor are they stored as other neurotransmitters are. They are synthesized inductively, on-demand at dendritic spines and somatodendritic compartments of postsynaptic neurons from which they diffuse in retrograde fashion across synapses to presynaptic membranes and where their effects are mediated by G-protein coupled cannabinoid receptor subtypes 1 (CB1) and 2 (CB2) or G-protein-coupled receptor 55 (GPR55). CB1 is the most abundant in the central nervous system, detectable as early as embryogenesis. CB1 receptor density during early adolescence increases in all areas of the central nervous system, following the general pattern of neurotransmitter receptor systems typical to this developmental period. In later adolescence, this increase in CB density is reversed so much so that adult striatal CB1 density falls to 50% [14].

What is peculiar to the eCB system during the adolescent phase of development, in addition to changes in CB1 density, are the changes in AEA and 2-AG synthesis. Both AEA and 2-AG are present from early blastocyst formation and then during embryogenesis, where the latter surges to higher levels during embryonic tissue differentiation. Synthesis of both neurotransmitters decreases significantly during adolescence in the hippocampus, hypothalamus, amygdala, and the nucleus accumbens. While in the dorsolateral prefrontal cortex (DPFC), however, the synthesis of AEA is significantly increased and continues to do so into adulthood (Figure 1) [15].

Another peculiar finding during adolescence is the vulnerability of the dorsal striatum to long-term depression during adolescence as demonstrated in animal studies. Here, CB1 activation directly modulates glutaminergic neurotransmission in an intermediary process initiated by upstream GABA neurotransmission [16]. It is important to note here the use of WIN 55,212-2, an exogenous CB1 receptor agonist used to induce LTD at the dorsal striatal complex of adolescent mice independent of upstream GABA neurotransmission. WIN 55,212-2 failed to induce LTD in the dorsal striatum of adult mice, suggesting two important points regarding the adolescent eCB system. The first is that changes in the eCB system during adolescence may be queued by GABA-induced synaptogenesis and that, in turn, glutaminergic LTD may occur via endocannabinoid depolarization-induced suppression of excitation. As such, eCB activation may be the nidal event in synaptic pruning in later adolescence [17,18]. In other areas of the CNS such as the amygdala, the presence of CB1 on presynaptic GABA neurons further demonstrates the role of the eCB in modulating GABAergic circuits. At the basolateral amygdala, CB1 depolarization suppresses GABA inhibitory postsynaptic currents (IPSCs) and, in this case, serves to reduce inhibitory GABA activity [19]. The second point concerns the implications for the use of exogenous CB1 agonists such as THCΔ9 and synthetic cannabinoids in modulating long-term neuroplastic changes, particularly in the dorsal striatum with chronic use in early adolescence. While further studies may be needed to understand the mechanisms, what is already clear about the eCB system in adolescence is that it is integral in the development of other neurotransmitter systems and that the frontal and striatal regions are especially vulnerable in the context of cognitive processing and emotional regulation.

Depression onset

**Figure 2:**
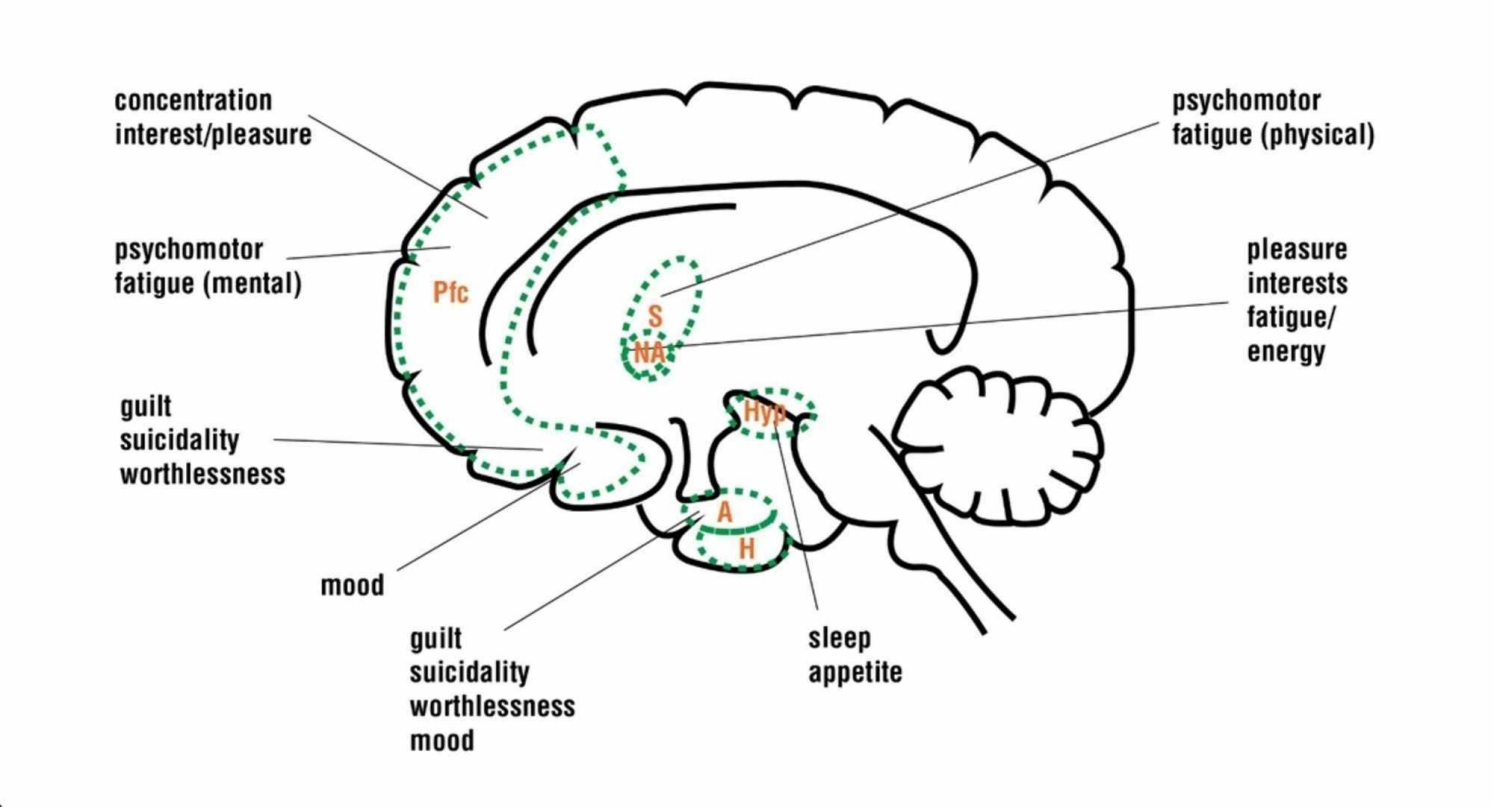
Neocortical and subcortical circuits associated with depression Pfc: prefrontal cortex; S: striatum; NA: nucleus accumbens; Hyp: hypothalamus; A: amygdala; H: hippocampus

Depression is a chronic relapsing disorder of complex multifactorial etiology characterized by dysregulation of mood, decreased emotional reactivity to positive external experience, decreased sexual motivation, rumination and, in severe cases, suicide. Depression onset is linked to dysregulated monoamine neurotransmitter signaling areas of the brain controlling effect, emotional reactivity, cognitive processing, and motivation (Figure 2) [20,21]. It is important to note that normal physiological eCB signaling is essential in promoting mood and that abnormal eCB signaling, which is a decrease or increase, has been shown to elicit depression-like phenotypes [22]. CB1 receptor blockade, for instance, results in a decrease in serotonin (5-HT) levels in the frontal cortices in animal models [23]. Upregulation of CB1 receptor-mediated guanosine triphosphate gamma-S protein (GTPꝩS) binding, on the other hand, maybe associated with human suicidality in depressed human subjects [24]. The eCB dysregulation resulting in either increased or decreased signaling as a result of THCΔ9 can potentially affect any neural circuit where CB1 is abundant. CB1 activation in the PFC, dorsal striatum, and oligodendrocytes are especially important in the discussion of depression emergence.

Anhedonia and reduced motivation

Acute cannabinoid exposure results in euphoria, reduced sensitivity to stimuli with negative emotional content, and heightened sensitivity to stimuli with positive emotional content. Heavy, chronic cannabis use in adolescence, however, may induce subtle changes in neural circuits leading to altered emotional reactivity and decreased social behavior in later life [25]. WIN-55,212-2 doses as low as 0.2mg/kg daily was associated with depressive symptoms in adolescent rodents, an effect associated with reduced monoaminergic (serotonin) neurotransmission [26]. Further animal studies demonstrated decreased social behavior later in life in both sexes when synthetic cannabinoids WIN55,212-2 or CP55,940 were inoculated during adolescence [27,28]. Both sexes were affected and no effect was observed in adult rodents. Exposure to THCΔ9 or WIN55,212-2 also induced passive coping strategy in forced swim test (a model for anhedonia), measured as sucrose preference or palatable food consumption (Table 1) [26,29]. Anhedonia resulting from cannabinoid exposure may be explained by the long-term depression that occurs at the level of the dorsal striatum [30]. The effects of chronic exposure seem to be dependent on the age of THCΔ9 exposure, dose, and chronicity [31]. Adolescent cannabis users who developed depressive symptoms including anhedonia are more likely to be using cannabinoids in escalating doses.

**Table 1:** Cannabinoid-CBR mediated changes in the development of depressive symptoms LTD: long-term depression; PFC: prefrontal cortex; FST: forced swim test; GAD: glutamic acid decarboxylase; GABA: gamma-aminobutyric acid; GLuN2B: glutamate N-methyl D-aspartate receptor type subunit 2B; GLuA: glutamate transport ATP-binding protein; THCΔ9: delta-9-tetrahydrocannabinol; CBR: cannabinoid receptor

Endocannabinoid effector	CBR-mediated effects
THCΔ9, WIN-55, 212-2	LTD in nucleus accumbens, decreased GABA PFC transmission, induction of passive coping strategy (FST model)
CP55 940	Decreased libido, decreased K+ evoked glutamate release, increased GluN2B, GLuA in PFC
THCΔ9	Decreased GAD 67 and basal GABA in PFC

In users who escalated cannabis dosing over time, disruptions in white matter tracts with or without compensatory aberrant connectivity in several circuits were associated with apathy, decreased motivation, anhedonia, and increased default mode network (DMN) activity. Reduced connectivity was observed between the medial PFC and the nucleus accumbens, white matter disruption in the frontolimbic tracts in fatty acid amide hydrolase (FAAH) C/C genotypes, and aberrant connectivity between the left orbitofrontal and the left parietal cortices [31-33]. THCΔ9 exposure profoundly affects cannabinoid receptor expression and G-protein coupling. CB1 density on exposure to THCΔ9 is significantly decreased in both sexes, but the effect was greater in female rats [34,35]. Decreased CB1 density in the PFC seems to be paralleled by decreased AEA levels and decreased CB1/G-protein coupling. A compensatory CB1/G-protein upregulation is observed after cannabis cessation. It is important here to reiterate that chronic exposure to cannabinoids during adolescence based on animal studies did not bring about similar changes in adult controls, indicating a specific vulnerability of cannabinoid receptor coupling in this age group [26].

By downregulating CB1 and AEA, exogenous cannabinoids may disrupt the normal neurodevelopmental processes characterizing adolescence: synaptogenesis, synaptic pruning, and white matter development [36]. This is especially important in lieu of the queuing function of eCB signaling on neurotransmitter systems and their purpose in consolidating white matter tracts and neuroplastic potentiation typical of developmental maturity. Cannabinoids in this regard not only disrupt the role the eCB has in plasticity, but they also interfere with glutamate signaling as well. THCΔ9 disrupts the normal sequencing of NMDA subunits throughout adolescent development [37-39]. NMDA receptor subunit 2B (GluN2B) is typically substituted for the GluN2A subtype throughout later adolescence so that GluN2A is more abundant in adulthood. In cannabis users, this switch from GluN2B to GluN2A does not occur [34]. Disruption of the normal neuroplastic changes that lead to maturity, especially in the prefrontal cortex and the nucleus accumbens, are especially significant to the trajectory and permanence of maladaptive social behavior, reduced motivation, and apathy associated with adolescent cannabis use.

Suicide

Suicide, an event that is attributable to major depression, is now the second most common cause of death in the adolescent age group [40]. Although human depression may be associated with increased eCB activity, exogenous cannabinoids have not been definitively associated with an increased risk of suicidality [1,24,41].

Rumination

Rumination is defined as a response state that hyper-focuses on distress symptoms and their perceived cause(s) and outcomes, which are usually negative. Rumination, though not a defining symptom of depression, is commonly associated with major depression and is indicative of depression onset, its severity, and duration of symptoms [42]. The DMN is the primary neural facilitator of the ruminating process, specifically in the dorsomedial PFC [43]. Early-onset cannabinoid use has been associated with hyperconnectivity within the DMN [44].

Genetic determinants

Genetic covariables, namely the endocannabinoid receptor (CNR) gene, dopamine, and 5-HT polymorphisms appear to influence the development of depressive symptoms in cannabinoid users significantly. Cannabis use over time appears to increase the risk for depressive symptoms in the presence of the short allele of the serotonin-transporter-linked polymorphic region (5-HTTLPR) [45]. Further data from genome-wide bivariate linkage analysis suggest that cannabis use and depression are co-heritable traits. A pleiotropic locus identified on chromosome 11q23.1-q23.2 comprises a gene sequence comprising nuclear cell adhesion molecule 1 (NCAM1), dopamine receptor D2 (DRD2), tetratricopeptide repeat domain 12 (TTC12), and ankyrin repeat and kinase domain containing 1 (ANKK1) [46]. While NCAM1 and DRD2 gene expression are associated with depressive symptoms independently, there is limited data on the functions of this cluster. Activity within the dopamine system may underlie the initiation of marijuana use, as well as the continuation and escalation of use. Besides DRD2 gene products, dopamine receptor D4 (DRD4) gene polymorphism is also independently linked to both substance abuse, including cannabis use and internalizing disorders [47]. The odds of being comorbid for depressive symptoms and marijuana use are about 2.5 times higher for youths carrying the ≥7R/≥7R genotype than youths who carry the <7R/<7R genotype, controlling for the effects of ethnicity, gender, age, violent victimization, and alcohol-related problems [48]. In addition to these genetic influences, data concerning CNR gene polymorphisms suggest susceptibility to mood disorders as well as cannabis dependence and addiction [49,50]. Data on the epigenetic mechanisms and the significance of the linkage between CNR, DRD4, and the NCAM1-TTC12-ANKK1-DRD2 loci are limited and further studies would be needed to elucidate how exogenous cannabinoids modulate their expression.

## Conclusions

While adolescent cannabinoid use does not appear to give rise to clinically overt or severe forms of major depression consistent with the fifth Diagnostic and Statistical Manual of Mental Disorders (DSM-V) criteria, genetically vulnerable individuals may suffer worsening of decrease in motivation and socialization as well as ruminating practices. Pleiotropic linkage of the NCAM1-TTC12-ANKK1-DRD2 locus and other genetic determinants including CNR and 5-HTTLPR may explain the strong co-occurrence of these depressive symptoms with cannabinoid use. These symptoms, and their trajectory from adolescence into adulthood, seem to have definable cannabinoid-mediated mechanisms such as dysregulated glutaminergic and GABA neurotransmitter circuits and reduced monoamine signaling.
